# Supplementary material for: Perception of an object’s global shape is best described by a model of skeletal structure in human infants
Source: eLife. 2022 May 25;11:e74943. doi: 10.7554/eLife.74943 (PMC9132572; doi:10.7554/eLife.74943)
Supplement: Transparent reporting form [file elife-74943-transrepform1.pdf]

eLIFE
1st Floor
24 Hills Road
Cambridge CB2 1JP, UK
P 01223 855340
W elifesciences.org
T @elife

eLife's transparent reporting form

We encourage authors to provide detailed information *within their submission* to facilitate the interpretation and replication of experiments. Authors can upload supporting documentation to indicate the use of appropriate reporting guidelines for health-related research (see EQUATOR Network), life science research (see the BioSharing Information Resource), or the ARRIVE guidelines for reporting work involving animal research. Where applicable, authors should refer to any relevant reporting standards documents in this form.

If you have any questions, please consult our Journal Policies and/or contact us: editorial@elifesciences.org.

Sample-size estimation

- You should state whether an appropriate sample size was computed when the study was being designed
- You should state the statistical method of sample size computation and any required assumptions
- If no explicit power analysis was used, you should describe how you decided what sample (replicate) size (number) to use

Please outline where this information can be found within the submission (e.g., sections or figure legends), or explain why this information doesn't apply to your submission:

Sample size for Experiment 1 was determined using an a priori power analysis with a hypothesized medium effect size ($d = 0.50$; $1 - \beta > .8$). For Experiment 2, we hypothesized that objects with the same coarse spatial relations would be more difficult to discriminate because of shape skeletons that were more similar to one another (compared to Experiment 1), leading to an attenuated effect of categorization. Accordingly, to retain adequate power, we tested 14 more infants than in Experiment 1, the exact number of which was determined according to a fully counterbalanced design. Importantly, we nevertheless find the same results in Experiment 2 when the sample size is subsampled ($n = 34$) to match that of Experiment 1.

Sample size information can be found in the Materials and Methods section under 'Participants.'

Replicates

- You should report how often each experiment was performed
- You should include a definition of biological versus technical replication
- The data obtained should be provided and sufficient information should be provided to indicate the number of independent biological and/or technical replicates
- If you encountered any outliers, you should describe how these were handled
- Criteria for exclusion/inclusion of data should be clearly stated
- High-throughput sequence data should be uploaded before submission, with a private link for reviewers provided (these are available from both GEO and ArrayExpress)

Please outline where this information can be found within the submission (e.g., sections or figure legends), or explain why this information doesn't apply to your submission:

There were two experiments in the current study. The second experiment provides a conceptual replication and extension of the first experiment.

Ten infants were excluded from the analysis in total (Experiment 1: 5 for fussiness and 1 because of equipment failure; Experiment 2: 3 for fussiness and 1 because of equipment failure). Exclusion criteria can be found Sample size information can be found in the Materials and Methods section under 'Participants.'

Statistical reporting

- Statistical analysis methods should be described and justified
- Raw data should be presented in figures whenever informative to do so (typically when N per group is less than 10)
- For each experiment, you should identify the statistical tests used, exact values of N, definitions of center, methods of multiple test correction, and dispersion and precision measures (e.g., mean, median, SD, SEM, confidence intervals; and, for the major substantive results, a measure of effect size (e.g., Pearson's r, Cohen's d)
- Report exact p-values wherever possible alongside the summary statistics and 95% confidence intervals. These should be reported for all key questions and not only when the p-value is less than 0.05.

Please outline where this information can be found within the submission (e.g., sections or figure legends), or explain why this information doesn't apply to your submission:

Infants' looking times were analyzed using two-sided paired samples t-tests (α = .05) and standard measures of effect size. Furthermore, to ensure that sample size decisions did not unduly influence the results, we also conducted non-parametric (Binomial tests) and Bayesian analyses. A Bayes factor (BF10) was computed for each analysis using a standard Cauchy prior (d = 0.707). A BF10 greater than 1 suggest is evidence that two distributions are different from one another, whereas a BF10 less than 1 suggests is evidence that two distributions are similar to one another (Rouder, Speckman, Sun, Morey, & Iverson, 2009).

General information on data analysis is provided at the beginning of the Results section. Modeling details are found in the Materials and Methods section.

(For large datasets, or papers with a very large number of statistical tests, you may upload a single table file with tests, Ns, etc., with reference to sections in the manuscript.)

Group allocation

- Indicate how samples were allocated into experimental groups (in the case of clinical studies, please specify allocation to treatment method); if randomization was used, please also state if restricted randomization was applied
- Indicate if masking was used during group allocation, data collection and/or data analysis

Please outline where this information can be found within the submission (e.g., sections or figure legends), or explain why this information doesn't apply to your submission:

NA

Additional data files ("source data")

- We encourage you to upload relevant additional data files, such as numerical data that are represented as a graph in a figure, or as a summary table
- Where provided, these should be in the most useful format, and they can be uploaded as "Source data" files linked to a main figure or table
- Include model definition files including the full list of parameters used
- Include code used for data analysis (e.g., R, MatLab)
- Avoid stating that data files are "available upon request"

Please indicate the figures or tables for which source data files have been provided:

Data files for every analyses have been made available on the open science framework (OSF). All modeling code will be made available on GitHub.
